# A Simple Pump-Free Approach to Generating High-Throughput Microdroplets Using Oscillating Microcone Arrays

**DOI:** 10.3390/mi15111365

**Published:** 2024-11-12

**Authors:** Erturan Yetiskin, Ilayda Erdem, Sinan Gucluer, Adem Ozcelik

**Affiliations:** 1 Graduate School of Natural and Applied Science, Aydin Adnan Menderes University, Aydin 09010, Türkiye; 2211500111@stu.adu.edu.tr; 2 Department of Mechanical Engineering, Aydin Adnan Menderes University, Aydin 09010, Türkiyesgucluer@adu.edu.tr (S.G.)

**Keywords:** droplet microfluidics, acoustofluidics, piezo-transducer, oil droplets

## Abstract

Droplet generation is crucial in various scientific and industrial fields, such as drug delivery, diagnostics, and inkjet printing. While microfluidic platforms enable precise droplet formation, traditional methods often require costly and complex setups, limiting their accessibility. This study introduces a simple, low-cost approach using an off-the-shelf unit and a 3D-printed reservoir. The device, equipped with a driver board, piezo-ring transducer, and a metal sheet with holes, generates oil-in-water (O/W) droplets with an average diameter of 4.62 ± 0.67 µm without external fluid pumps. Its simplicity, cost-effectiveness, and scalability make it highly suitable for both lab-on-chip and industrial applications, demonstrating the feasibility of large-scale uniform droplet production.

## 1. Introduction

Droplet generation is a pivotal process in numerous scientific and industrial applications, ranging from drug delivery and diagnostics to inkjet printing and food processing [1,2,3]. These applications rely heavily on precision and control over droplet formation, as droplet size, uniformity, and composition can significantly influence both the efficacy and efficiency of the processes involved. The field of microfluidics has catalyzed the development of a wide array of platforms capable of generating droplets with diverse types and compositions [4,5,6,7]. Microfluidic-based droplet generation offers a flexible, scalable, and reproducible platform, making it an invaluable tool for researchers across disciplines, from biomedicine to chemical engineering [8,9,10].

While droplet microfluidics offer a versatile platform, traditional microfluidics methods of droplet generation often rely on sophisticated equipment and fabrication processes that increase costs and limit accessibility, particularly in resource-constrained settings [11,12]. This reliance on high-precision equipment not only elevates the cost but also restricts accessibility, especially in locations without access to clean-room facilities or expensive fluid control systems. For instance, conventional methods like photolithography and soft lithography involve time-intensive steps and specialized infrastructure, creating an accessibility barrier for many researchers. As a result, there is a growing interest in developing alternative, low-cost fabrication methods for droplet-generating devices [12,13]. However, these alternatives often still depend on external fluid control units, limiting their portability and adaptability for diverse applications beyond specialized lab environments.

Microfluidic platforms present unique capabilities in terms of fluid manipulation on a small scale by offering precision and integration for on-chip applications [14,15,16,17]. The ability to handle minute liquid volumes with a high degree of control also makes microfluidic tools suitable for droplet formation, which can be achieved through various configurations [18,19,20,21,22,23,24]. The most common approaches to microfluidic droplet generation involve flow-focusing devices and T-junctions, which utilize controlled flow rates and microscale channel geometries to create single- and multi-layer droplets, as well as droplet-encapsulated cargoes [13,25,26,27,28]. These designs allow for precise control over droplet formation, enabling the encapsulation of various materials, making them suitable for applications that require highly uniform droplet sizes and contents.

Beyond flow-focusing methods, external mechanisms such as surface acoustic waves (SAWs) have been incorporated to control droplet content and behavior through fluid actuation [20,29,30,31,32,33]. Acoustic actuation methods, including droplet ejection from liquid puddles via acoustofluidic and digital microfluidics, have expanded the capabilities of microfluidic droplet systems [34,35,36,37,38]. However, microfluidic droplet generation devices are typically fabricated in clean-room facilities equipped with high-cost equipment, which limits accessibility for researchers worldwide. To overcome these obstacles, alternative fabrication techniques, such as 3D printing and laser prototyping, have been developed to create lower-cost devices for droplet generation [39,40,41,42,43]. While such approaches make microfluidic droplet generation platforms more accessible, they still often require external fluid control units. This reliance highlights the need for a low-cost, scalable, and user-friendly device for generating microscale droplets suitable for both on-chip and industrial applications.

Herein, we present a very simple and efficient approach for droplet generation using a low-cost, off-the-shelf unit attached to a 3D-printed fluid reservoir. This unit comprises a driver board, a piezo-ring transducer, and a thin metal sheet with an array of holes. This droplet generation approach does not require any external fluid pumping units and works by placing the device in any vessel, such as a Petri dish, containing one of the phases of the droplet system to start generating droplets. Based on the specifications of the off-the-shelf component, the presented device generated oil-in-water (O/W) droplets with an average diameter of 4.62 ± 0.67 µm. Thanks to its simplicity, cost-effectiveness, and dexterity, this approach holds significant promise for lab-on-chip applications and is highly scalable for industrial processes.

## 2. Materials and Methods

**Figure 1:**
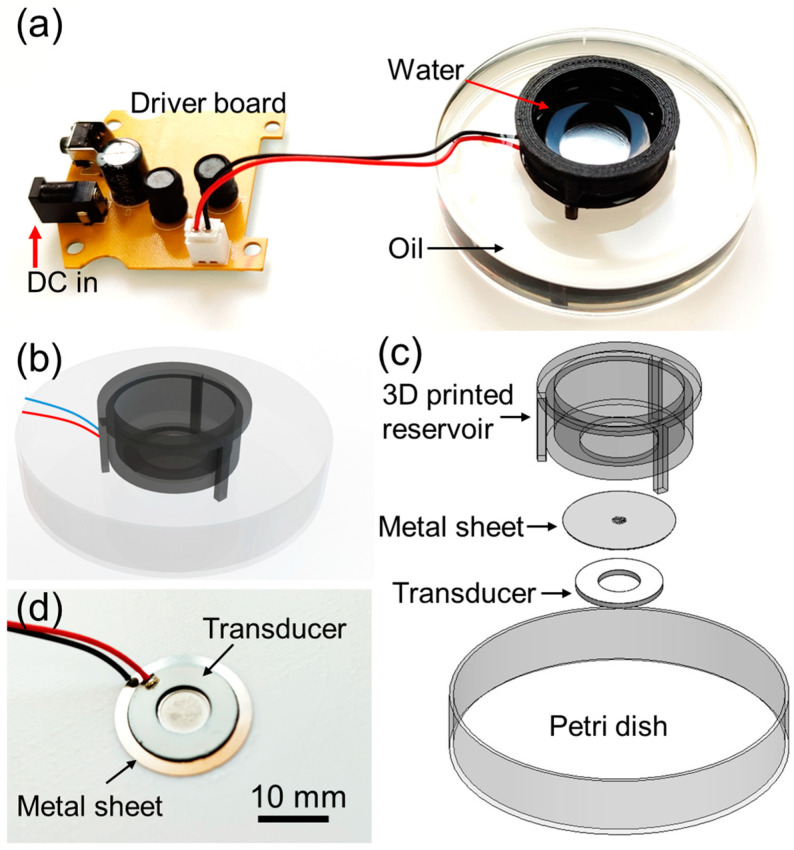
Device details. (**a**) Picture of the control board and the piezo-transducer assembly placed in an oil-filled Petri dish. (**b**) Schematic depiction of the piezo-transducer and 3D-printed part assembly. (**c**) Exploded view of the device. (**d**) Piezo-transducer and metal sheet.

The droplet generation device depicted in Figure 1 comprises a 3D-printed reservoir mounted on a mini tripod, an off-the-shelf unit (Model 113 kHz, TaiMi, Shenzhen, China) featuring a control board, a piezo-ring transducer with a resonance frequency of 113 kHz, and a thin metal sheet perforated with an array of conical holes. A consumer-grade 3D printer (Ender 3, Creality, Shenzhen, China) is used for printing the parts with a standard PLA filament with the following parameters: infill is 100%, print speed is 60 mm/s, layer height is 0.2 mm, nozzle temperature is 210 °C, and print bed temperature is 60 °C. The piezo-ring transducer and the metal sheet are pre-bonded concentrically by the manufacturer, which is required for the operation of the device. The driver board is powered by a 12 V DC power input and can drive the 113 kHz transducer. A three-dimensionally printed reservoir is attached to the piezo-ring assembly using a silicone sealant (APEL, Beta Chemicals, Istanbul, Türkiye), as shown in Figure 1. For our demonstration of oil-in-water (O/W) droplets, larger-diameter holes of the conic-shaped hole array on the metal sheet face up towards the 3D-printed reservoir. For water-in-oil droplets, the direction can be reversed. For the oil phase, generic sunflower seed oil is used, and for the water phase, deionized water and 2% Tween 20 (Sigma Aldrich, St. Louis, MO, USA) are used. 

Droplets are imaged using an inverted optical microscope equipped with 10×, 20×, 40×, and 60× Plan Fluor objective lenses, with an HD camera (OX.2053-PLPH, Euromex, Arnhem, The Netherlands). Pictures of the hole arrays on the metal sheet are imaged using an inverted metal microscope (XJP-6 A, Soif, Guangzhou, China) equipped with a CMOS camera (MS60, M-Shot, Guangzhou, China). For device operation, the reservoir-transducer assembly is filled with di water mixed with 2% Tween 20 and placed in a Petri dish filled with the oil. Droplet sizes are analyzed using Image J software.

## 3. Results and Discussions

### 3.1. Working Mechanism

**Figure 2:**
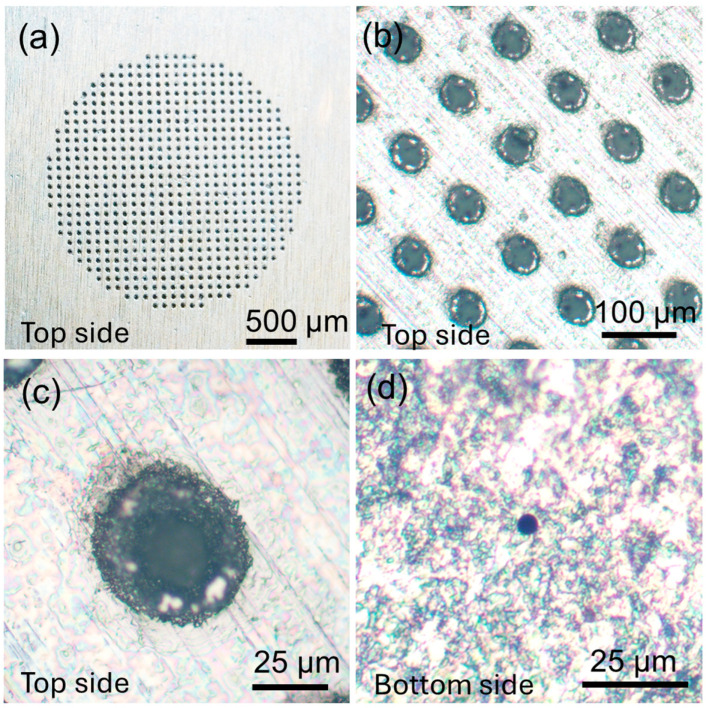
(**a**) Microcone array in the center of the metal sheet. Closer view of (**b**) the array and (**c**) a single hole from the top side. (**d**) A single hole from the bottom side.

**Figure 3:**
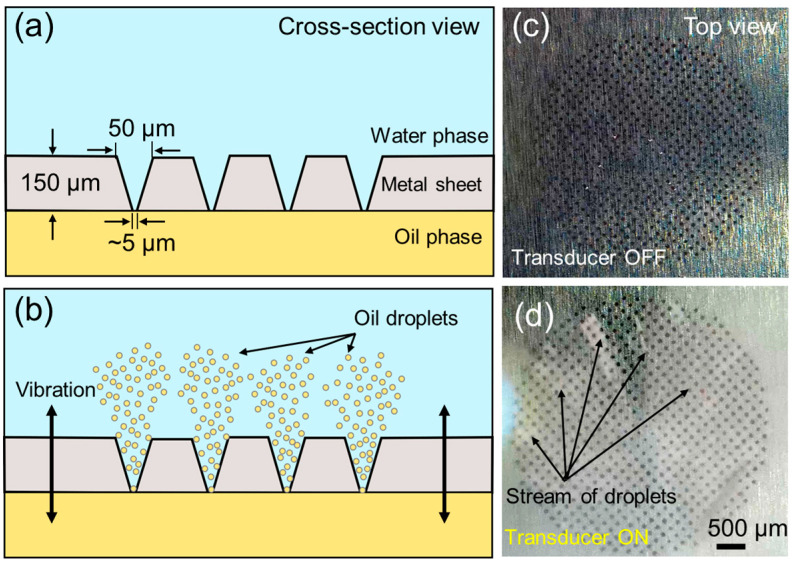
Working principle of the droplet generation. (**a**) Schematic illustration of the cross-section of the device showing water and oil phases, and dimensions of the cones (not drawn to scale). (**b**) When the transducer is actuated, oil droplets are ejected into the water phase. (**c**) Top view of the microcone array. (**d**) Generation of oil droplets in the water phase observed from the top view.

The presented droplet generation approach relies on vibration of the conical hole array on the metal sheet of 150 µm thickness, which features 50 µm diameter holes on the one side (top side) and approximately 5 µm diameter holes on the other side (bottom side) of the sheet, as shown in Figure 2. The total number of holes on the metal sheet is 608, as shown in Figure 2a. A closer inspection of the holes reveals the roughly conic shapes in Figure 2b,c. A smaller hole is shown on the bottom side of the sheet in Figure 2d. For generating O/W droplets, oil and water phases are introduced to the two faces of the metal sheet, as shown in Figure 3a. When the transducer is actuated at 113 kHz, these microcones oscillate back and forth and, in turn, eject oil droplets through the smaller-sized holes to the larger ones into the water phase, as schematically illustrated in Figure 3b. The top views of the metal sheet, where larger-diameter holes are exposed in the water phase, are also shown in Figure 3c,d to demonstrate the generation of droplets that are emerging as a white stream when the transducer is actuated (see Supplementary Video S1). To further understand the performance of the piezoelectric transducer, we measured its frequency response using a vector network analyzer (Supplementary Figure S1). The transducer exhibited a strong resonance at 113 kHz and a weaker response near 140 kHz. While we focused on this frequency in our current study, we recognize the potential to explore higher frequencies, such as those in the MHz range, which could enable different droplet size distributions and generation efficiencies.

### 3.2. Droplet Formation and Characterization

**Figure 4:**
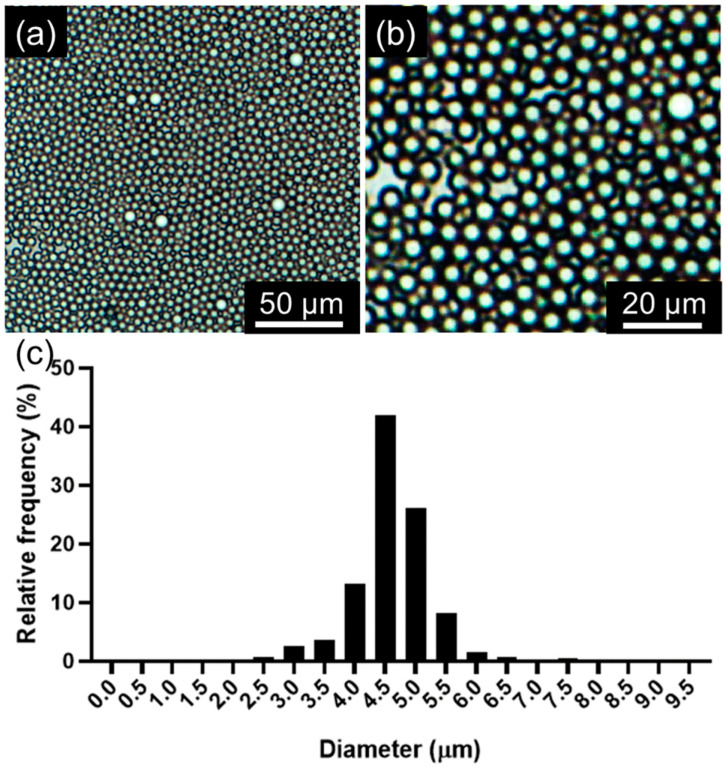
Characterization of droplet sizes. (**a**) A wide-view image of generated oil droplets. (**b**) Zoomed view of the droplets. (**c**) The distribution of the oil droplet sizes is shown as relative frequency versus droplet diameter plot.

O/W droplets are prepared using the configuration shown in Figure 3a. The average diameter of the droplets is 4.62 µm with a standard deviation of 0.67 µm. Figure 4 shows images and the size distribution of the droplets, closely matching the 5 µm diameter of the smaller holes. This consistency highlights the crucial role of the conical hole design in ensuring uniform droplet size. Minor variations in size are due to manufacturing inconsistencies, but overall, the device effectively produces uniform droplets. This uniformity is vital for applications like pharmaceuticals, where consistent droplet size impacts efficacy. While the current system generates droplets with an average diameter of 4.62 ± 0.67 µm, we recognize the need for larger droplets, particularly for biological applications. To address this, we have initiated discussions with manufacturers to create custom-designed hole arrays with larger dimensions. This approach will allow us to tune droplet sizes in the range of 50 µm and beyond, which would significantly expand the range of potential applications, such as in cell encapsulation and drug delivery.

As shown in Figure 3b, oil droplets are ejected from each microcone hole via oscillation of the metal sheet at 113 kHz. Assuming each oscillation produces one droplet, each microcone can generate approximately 113,000 droplets per second. With 608 microcone holes, the device can theoretically produce over 68 million droplets per second. This number can be significantly increased with custom fabrication of more microcones and by using multiple transducer arrays in a larger vessel, enabling industrial-scale droplet production within a few micrometers in diameter. To evaluate the practical throughput of the device, we conducted a series of experiments to approximately quantify the throughput of the device, normalizing droplet counts to droplets per second (Supplementary Figure S2). During a 10 s run, the device produced approximately 18.24 ± 8.7 million droplets per second, which increased significantly to over 42 million droplets per second at 60 s. This increase in throughput over time may be due to the release of air bubbles or other impurities trapped in the microcone arrays during initial operation. Although the throughput plateaued after 60 s, the results demonstrate that the device can generate a high number of droplets per second, making it suitable for high-throughput applications.

**Figure 5:**
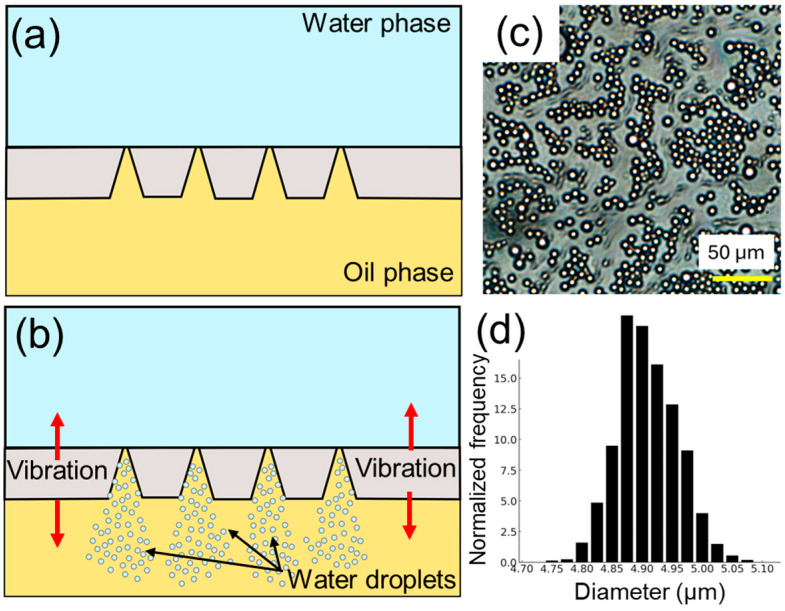
Generation of water-in-oil droplets by reversing the transducer orientation, allowing stabilized water droplets to sink into the oil phase, ensuring continuous droplet formation without interference. (**a**) Schematic illustration of the cross-section of the device showing water and oil phases. (**b**) When the transducer is actuated, water droplets are ejected into the oil phase. (**c**) Generated water droplets. (**d**) Size distribution of the generated water droplets.

To expand the system’s versatility, we adapted it to generate water-in-oil droplets by reversing the orientation of the transducer (Figure 5). In this configuration, water droplets were ejected downward into an oil phase containing Span 80, which stabilized the droplets. Simply exchanging the positions of oil and water phases was ineffective, as water droplets—being denser than oil—accumulated on the metal membrane, obstructing continuous droplet formation. Reorienting the transducer allowed the conical hole array to face downward, enabling water droplets to settle at the bottom of the oil phase. This approach produced water droplets with a size distribution similar to the original oil-in-water droplets, demonstrating the system’s adaptability for various droplet generation needs, including applications requiring stable water-in-oil systems.

## 4. Conclusions

This study introduces a scalable and efficient droplet generation method using oscillating conical hole arrays on a 150 µm thick metal sheet with 608 holes, each tapered from 50 µm on the top side to about 5 µm on the bottom. Actuation at 113 kHz allows the transducer to eject oil droplets into the water phase, producing droplets with an average diameter of 4.62 µm and a standard deviation of 0.67 µm. This method’s scalability, simplicity, and cost-effectiveness make it ideal for both research and industrial applications, integrating easily into existing workflows without major modifications. It consistently generates uniform droplets at high throughput, surpassing conventional microfluidic systems and offering precise control necessary for targeted drug delivery, diagnostics, and large-scale material synthesis.

The platform also holds promise for encapsulating biological cells or particles within droplets, which could have important implications for biomedical applications such as cell culture, diagnostics, and drug delivery. While encapsulation was not explored in the current study, we plan to investigate this potential in future work, with specific attention to encapsulating different cell types such as bacteria or immune cells.

The approach’s capacity to produce over 60 million droplets per second can be further enhanced by expanding the number of conical holes and integrating multiple transducers, meeting industrial-scale demands. By eliminating the need for complex flow controls and clean-room fabrication, it reduces operational costs and technical barriers, enhancing accessibility. Future improvements, such as optimizing the conical array design and expanding fluid compatibility, could further extend its utility, positioning this technology as a transformative solution in droplet microfluidics. This robust and adaptable method addresses the need for precise, high-throughput droplet generation, promising significant advancements across applications requiring uniform and efficient droplet production.

Building on the current findings, future work will focus on expanding the capabilities of the device by exploring custom transducers with varying resonance frequencies, different microcone array dimensions, and higher-frequency actuation. These investigations will enable further optimization of droplet size and throughput, broadening the device’s applicability in both research and industrial settings.

## Data Availability

Data are available within reasonable request from the corresponding author.

## Supplementary Materials

The following supporting information can be downloaded at https://www.mdpi.com/article/10.3390/mi15111365/s1, Video S1: Droplet generation. Figure S1: Characterization of the frequency response of the transducer. Figure S2: Characterization of the throughput of the device.
