# Supplementary material for: A Simple Pump-Free Approach to Generating High-Throughput Microdroplets Using Oscillating Microcone Arrays
Source: Micromachines (Basel). 2024 Nov 12;15(11):1365. doi: 10.3390/mi15111365 (PMC11597012; doi:10.3390/mi15111365)
Supplement: Supplementary file 1 [file micromachines-15-01365-s001.zip › micromachines-3318458-supplementary.pdf]

A simple pump-free approach to generating high-throughput microdroplets using oscillating microcone arrays

Erturan Yetiskin [1,2], Ilayda Erdem [2], Sinan Gucluer [2] and Adem Ozcelik [2,*]

[1] Graduate School of Natural and Applied Science, Aydin Adnan Menderes University, Aydin, Turkiye; 2211500111@stu.adu.edu.tr

[2] Department of Mechanical Engineering, Aydin Adnan Menderes University, Aydin, Turkiye; 2311500108@stu.adu.edu.tr, sgucluer@adu.edu.tr, aozcelik@adu.edu.tr

* Correspondence: aozcelik@adu.edu.tr

Supplementary Information

Supplementary Figures

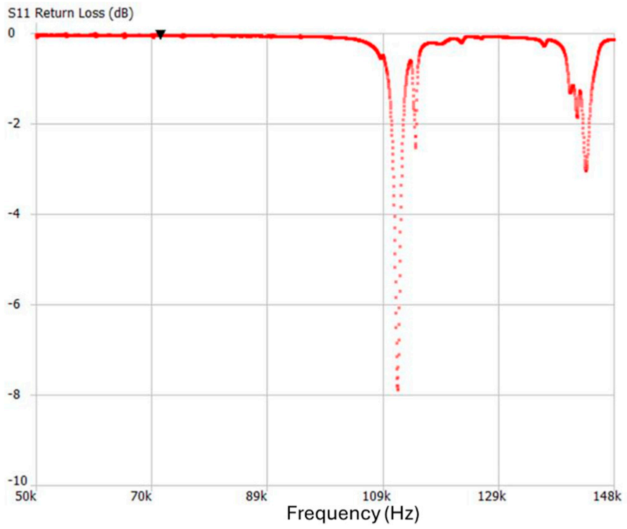

Figure S1. Characterization of the frequency response of the transducer.

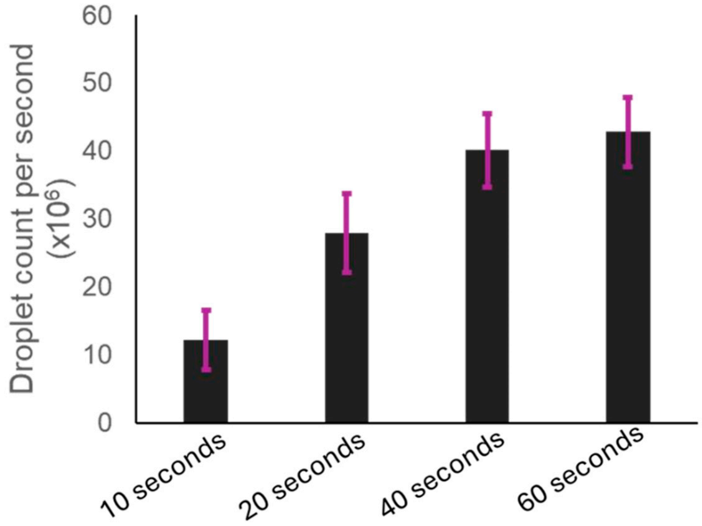

Figure S2. Characterization of the throughput of the device. To evaluate the practical throughput of the device, we conducted a series of experiments to approximately quantify the throughput of the device, normalizing droplet counts to droplets per second (Supplementary Figure S3). For this, 1 mL of water medium was added into the 3D-printed top reservoir and sample fluids were collected from each experiment after homogenizing the water phase by gentle shaking. We ran the droplet generation device for 10 seconds, 20 seconds, 40 seconds, and 60 seconds, and counted the generated droplets using a hemocytometer glass slide. We then normalized the droplet counts to per second for each condition. Each experiment was repeated with three different devices. During a 10-second run, the device produced approximately 18.24 ± 8.7 million droplets per second, which increased significantly to over 42 million droplets per second at 60 seconds. This increase in throughput over time may be due to the release of air bubbles or other impurities trapped in the microcone arrays during initial operation. Although the throughput plateaued after 60 seconds, the results demonstrate that the device can generate a high number of droplets per second, making it suitable for high-throughput applications.

Supplementary Videos

A video from the top view is taken which shows the generation of droplets once the transducer is turned on.
